# Universal tumor screening for lynch syndrome on colorectal cancer biopsies impacts surgical treatment decisions

**DOI:** 10.1007/s10689-022-00302-3

**Published:** 2022-06-23

**Authors:** Jennifer Vazzano, Jewel Tomlinson, Peter P. Stanich, Rachel Pearlman, Matthew F. Kalady, Wei Chen, Heather Hampel, Wendy L. Frankel

**Affiliations:** 1 Department of Internal Medicine, The Ohio State University Wexner Medical Center, Columbus, OH USA; 2 Department of Pathology, The Ohio State University Wexner Medical Center, Optometry Clinic and Health Science Faculty Office Building, 1664 Neil Avenue, Suite 6100, Columbus, OH 43210 USA; 3 Department of Surgery, The Ohio State University Wexner Medical Center, Columbus, OH USA; 4 Department of Medical Oncology and Therapeutics Research, City of Hope National Medical Center, Duarte, CA USA

**Keywords:** Universal tumor screening, Lynch syndrome, Colorectal cancer, Hereditary colorectal cancer

## Abstract

Universal tumor screening (UTS) for Lynch syndrome (LS) on colorectal cancer (CRC) can be performed on biopsies or resection specimens. The advantage of biopsies is the chance to provide preoperative genetic counseling/testing (GC/T) so patients diagnosed with LS can make informed decisions regarding resection extent. We evaluated utilization of UTS on biopsies, percentage of patients with deficient mismatch repair (dMMR) who underwent GC/T preoperatively, and whether surgical/treatment decisions were impacted. We performed a retrospective review of medical records to assess CRC cases with dMMR immunohistochemical staining from 1/1/2017 to 2/26/2021. 1144 CRC patients had UTS using MMR immunohistochemistry; 559 biopsies (48.9%) and 585 resections (51.1%). The main reason UTS was not performed on biopsy was it occurred outside our health system. 58 (5%) of CRCs were dMMR and did not have *MLH1* promoter hypermethylation (if MLH1 and PMS2 absent). 28/58 (48.3%) of dMMR cases were diagnosed on biopsy. Of those 28, 14 (50%) eventually underwent GC/T, and 7 (25%) had GT results prior to surgery. One of the 7 had incomplete documentation of results affecting their treatment plan. Of the remaining 6 with complete documentation, 5 underwent surgery and one was treated with immunotherapy only. Three patients elected a more extensive surgery. 6/28 (21.4%) dMMR patients identified on biopsy made an informed surgical/treatment decision based on their dMMR status/LS diagnosis. When applied, UTS on biopsy followed by genetic counseling and testing informs surgical decision-making. Process and implementation strategies are in place to overcome challenges to more broadly optimize this approach.

## Introduction

Lynch syndrome (LS) is the most common hereditary predisposition to colorectal cancer (CRC) and is caused by a pathogenic germline variant in one of the DNA mismatch repair (MMR) genes (*MLH1, MSH2, MSH6, PMS2)* or deletions in the 3’ region of *EPCAM* [1]*.* A previous study by Pearlman et al*.* found that LS has a remarkably high prevalence, affecting one out of every 25 patients diagnosed with CRC [2].

Microsatellite instability and/or loss of expression of one or more MMR proteins are hallmark characteristics of LS-associated tumors. Tumor screening using immunohistochemical (IHC) staining for expression of the four MMR proteins allows for identification of patients with MMR deficient (dMMR) tumors. Patients with dMMR tumors not explained by *MLH1* promoter hypermethylation should be referred for genetic counseling and discussion of confirmatory germline genetic testing for LS [3].

Historically, patients at increased risk of LS were identified through analysis of family history using criteria such as the Amsterdam Criteria and Bethesda Guidelines. However, studies have shown that these clinical criteria often miss patients with LS. One study found that limiting tumor analysis to patients who met the Bethesda Criteria failed to identify one in four cases of LS [4]. Another method for identifying patients at increased risk of LS is universal tumor screening (UTS). UTS involves screening all newly diagnosed CRC patients using MSI or IHC testing. UTS for LS on CRC is recommended by the National Society of Genetic Counselors (NSGC), National Comprehensive Cancer Network (NCCN), the U.S. Multi-Society Task on Colorectal Cancer, the American Gastroenterological Association, the American College of Gastroenterology, the Evaluation of Genomic Applications in Practice and Prevention (EGAPP) Working Group [5, 6] and the American Society of Colon and Rectal Surgeons [7]. UTS can be performed on diagnostic colonoscopy biopsies or on resected surgical specimens with equivalent results [8].

Colorectal cancer patients with LS have an increased risk of metachronous CRC [1]. The range of risk for metachronous CRC with segmental resection is 12–43%, and for extended colectomy is reduced to 0–18% [9]. As a result, some professional organizations including the American Society of Colon and Rectal Surgeons [7] recommend that individuals with LS who develop CRC should have subtotal or total colectomy instead of segmental resection to reduce the risk for a second primary CRC. Diagnosing LS on biopsies rather than resections potentially allows for preoperative genetic counseling/testing (GC/T) and informed surgical decision-making regarding the extent of colectomy. Our study aimed to analyze UTS on biopsy specimens and the impacts on preoperative diagnosis of LS and surgical decision-making.

## Methods

We performed a retrospective review of all CRC cases from the date our institution changed the UTS for LS protocol to include MMR IHC staining on CRC biopsies rather than resections wherever possible. As such, we reviewed all CRC cases from January 1, 2017 to February 26, 2021. Electronic medical records were reviewed for patients with dMMR, as defined by abnormal IHC. For tumors with absence of MLH1 and PMS2, *MLH1* hypermethylation or *BRAF* p.V600E results were assessed to determine what proportion of the patients needed referral to cancer genetics to rule out LS (those with evidence of hypermethylation were not routinely referred). Records were then further reviewed to note UTS for LS timing, referral to genetics, genetic test results, preoperative discussions, and surgical procedures/treatment plans. A Fisher’s exact t-test was used to compare the number of patients that had total colectomies in the group of patients that had a dMMR CRC identified with MMR IHC on biopsies and had colectomy (5/20) vs. the group of patients that had a dMMR CRC identified with MMR IHC on resections (1/30).

## Results

1144 consecutive CRC patients had UTS for LS using IHC staining for the four MMR proteins during the study period. MMR IHC was performed on a biopsy specimen for 559 patients (48.9%), while the other 585 (51.1%) had MMR IHC performed on their surgical resection specimen. Fifty-eight (5%) of the CRCs were dMMR as determined by loss of MSH2, MSH6, PMS2, or MLH1 without evidence of *MLH1* promoter hypermethylation. Of these 58 cases, 28 (48.3%) were diagnosed on biopsy rather than surgical resection allowing for the possibility of genetic referral and testing prior to surgery.

While 14 of the 28 (50%) underwent GC/T at some point in their care, only 7 (25%) had information about their germline status available for surgical decision-making (Table 1; cases 4, 5, 6, 17, 25, 27, and 28). Of these 7, 4 underwent GC/T prior to surgery and 3 had a previously known LS diagnoses. Of the 4 patients who underwent GC/T prior to surgery, 1 of them received neoadjuvant chemotherapy. There were several reasons why patients in our study were not seen for genetic counseling prior to surgery. Reasons included: patient lost to follow-up (n = 1), patient declining the appointment or not scheduling (n = 4), patient not referred (n = 3), patient with emergent need for surgery due to perforation (n = 2), patient receiving chemotherapy only (n = 1), patient receiving palliative care (n = 2), or death (n = 1). Three patients had GC/T at an outside institution.

**Table 1:** Demographic data of deficient mismatch repair colorectal carcinoma cases diagnosed on biopsy

	Age	Gender	Race	Tumor location	Immunohistochemical results	Treatment	Genetic status
1	62	Male	White	Right (ascending) colon	MSH6 absent	No surgery, palliative care	MSH6 mutation
2	31	Male	White	Transverse colon	MSH2 & MSH6 absent	None, chemo only	MSH2 mutation
3	50	Male	AA	Hepatic flexure	MSH6 absent	Laparoscopic right partial colectomy	MSH6 mutation
4	29	Male	White	Colon, descending	MLH1 & PMS2 absent	Total colectomy (larger surgery)	LS diagnosis already established
5	32	Male	White	Colon	PMS2 absent	Extended right hemicolectomy (smaller surgery elected)	PMS2 mutation
6	60	Male	White	Colon	PMS2 absent	Total (completion) colectomy (larger surgery due to previous right hemicolectomy and previous LS diagnosis)	PMS2 mutation
7	62	Male	White	Transverse colon	MSH2 absent	No surgery	LS diagnosis already established
8	79	Female	White	Splenic flexure	MLH1 & PMS2 absent	No surgery	Unknown, deceased
9	56	Male	AA	Colon	MLH1 & PMS2 absent	Open extended right hemicolectomy	Unknown
10	46	Male	White	Cecum and proximal transverse colon	MLH1 & PMS2 absent	Extended right hemicolectomy, en bloc resection abdominal wall and duodenum	Unknown
11	36	Male	White	Liver, colorectal origin	PMS2 absent	Small intestine, partial enterectomy	Unknown, deceased
12	43	Male	AA	Rectum	MSH2 absent	Neoadjuvant therapy, Rectosigmoid colon, laparoscopic low anterior resection	Unknown
13	42	Male	White	Liver met, colorectal origin	MLH1 & PMS2 absent	No surgery, palliative chemotherapy	Unknown
14	87	Female	White	Duodenum	MLH1 &PMS2 absent	Pancreaticoduodenectomy	Unknown
15	57	Female	White	Splenic flexure	MLH1 & PMS2 absent	Left colon, laparoscopic partial colectomy	Unknown
16	76	Male	White	Rectum	MSH2 absent	Neoadjuvant therapy, Partial laparoscopic colectomy	MSH2 mutation
17	43	Female	White	Right colon	MLH1 & PMS2 absent	Partial colectomy (surgical note mentions LS but not in relation to surgical plan)	MLH1 mutation
18	57	Male	White	Right (ascending) colon	PMS2 absent	Right colon, laparoscopic partial colectomy	Unknown
19	49	Female	White	Cecum	MSH2 & MSH6 absent	Partial colectomy	Unknown, deceased
20	78	Female	White	Small bowel	PMS2 absent	No surgery, deceased	Unknown, deceased
21	58	Male	White	Hepatic flexure	MSH2 & MSH6 absent	Laparoscopic extended right hemicolectomy	MSH2 mutation
22	56	Female	AA	Colon	MSH2 & MSH6 absent	Right hemicolectomy	MSH2 mutation
23	70	Male	White	Right colon/hepatic flexure	PMS2 absent	Laparoscopic partial colectomy	PMS2 mutation
24	64	Male	AA	Cecum and transverse colon	MLH1 & PMS2 absent	Right hemicolectomy	Negative
25	70	Male	White	Descending colon	PMS2 absent	Completion colectomy (larger surgery due to LS)	PMS2 mutation
26	68	Female	White	Colon	PMS absent	Neoadjuvant therapy, Ileum and colon, Colectomy	Unknown
27	39	Male	White	Colon	MSH6 absent	No surgery, immunotherapy	MSH6 mutation
28	42	Male	AA	Colon	MSH2 absent	Total colectomy (larger surgery due to LS)	MSH2 mutation

Of the 7 patients who had information about their germline status available for surgical decision-making, this information was included in the preoperative decision-making and clinical notes for 6 patients; for the one patient not included, there was incomplete documentation of their results impacting the surgical/treatment plan. Patient ages ranged from 29 to 70 (29, 32, 39, 42, 43, 60, and 70). Of the 6 patients with complete documentation, one patient did not have surgery and is on immunotherapy because of stage IV disease (case 27, Table 1). This treatment decision was based on the dMMR tumor, not LS status. For patients who had available information, it was utilized to guide treatment in all 6 of 6 patients. Of the 5 patients who had surgery, three patients had a more extensive colectomy due to their LS diagnosis (Cases 4, 25, 28, Table 1), while two elected a less extensive surgery after an informed risk/benefit discussion with their surgeon (Cases 5 and 17, Table 1).

Overall, 6 of 28 (21.4%) patients with dMMR CRC who underwent preoperative biopsy screening made an informed surgical or treatment decision based on their dMMR status/diagnosis of LS prior to resection. Of the remaining 22 patients: 2 had no surgery, 1 had no surgery due to death, 2 had no surgery and opted for palliative care only, 1 had no surgery and opted for chemotherapy only, 12 patients with colon cancer and 1 patient with rectal cancer underwent segmental resections, 1 patient with duodenal cancer had a pancreaticoduodenectomy, 1 patient had a partial enterectomy of the small intestine for metastatic cancer of colorectal origin, and 1 had total abdominal colectomy and ileorectal anastomosis (Table 1). Notably, when comparing these patients to the 30 patients that had dMMR CRC with MMR IHC testing done on their resections (post-surgery), we found that there were more patients that received total colectomies in this group (5 patients) than in the group that had their MMR IHC done on their postoperative resections (1 patient) (p = 0.03, Table 2).

**Table 2:** Surgical procedures performed for deficient mismatch repair colorectal carcinoma cases with mismatch repair protein immunohistochemistry performed on resection (postoperative) vs. biopsy (preoperative).

dMMR cases with MMR IHC performed on resections (#)	dMMR cases with MMR IHC performed on biopsies (#)
Right colectomy with en bloc removal of abdominal wall (1)	No surgery (6)
Segmental resections (including low anterior resection) (26)	Segmental resections (15)
Right hemicolectomy and en bloc small intestine (1)	Pancreaticoduodenectomy (1)
Ileocolectomy with appendectomy (1)	Partial enterectomy for metastatic colorectal carcinoma (1)
Total colectomy (1)	Total colectomy (5)
**Total cases (30)**	**Total cases (28)**

*dMMR* deficient mismatch repair, *MMR IHC* mismatch repair protein immunohistochemistry

## Discussion

Universal tumor screening for CRC and other tumors is essential for identifying dMMR and screening for LS. Studies have shown that UTS of all CRC cases for LS by analysis of microsatellite instability or IHC for MMR proteins is the most sensitive [10] and the most cost‐effective strategy for diagnosing LS [11, 12]. These studies, together with others [4, 13], have shown that UTS is efficient, sensitive, and cost-effective. UTS can expand the patients’ treatment options including immunotherapy and when LS is diagnosed in someone with CRC, their relatives can undergo cascade testing and begin proper intensive surveillance to prevent cancer. Previous studies have not specifically addressed the effect of UTS using preoperative biopsies rather than resections for surgical decision-making. Immunohistochemistry has been shown to work as well on biopsies as resections for the MMR proteins [8, 14, 15]. Internal positive controls were used in all specimens with special attention to the staining intensity of the tumor nuclei in comparison to the controls. The internal positive controls included background lymphocytes, stromal cells and benign epithelium (if present) [3]. We found that the programmatic approach of UTS on biopsies can be successful, but there is room for improvement in the process. We found that 21.4% of patients with dMMR CRC who underwent preoperative biopsy testing made an informed surgical or treatment decision based on their dMMR status/LS diagnosis prior to undergoing resection.

We found several barriers in the process that led to the low number of patients who were able to make an informed pre-operative decision. First, there were many cases without a biopsy available to screen at our institution before resection. The primary reason for this is challenges in obtaining tumor samples from patients coming into our healthcare system for treatment from outside institutions. Most pathology is reviewed prior to additional surgery at our institution, but not all cases are received, and tissue blocks are not submitted in many instances so MSI or IHC cannot be completed. The treating physician can request review of all biopsy material prior to additional treatment. If tissue blocks are not received for UTS, they can be requested by the treating physician or pathologist. Additionally, there are some instances when UTS on a biopsy is not possible, such as colon perforation requiring urgent surgery (this accounted for two patients in our cohort).

The success of UTS is dependent on patients receiving the screening results with subsequent pursuit of genetic counseling and germline genetic testing in a timely manner. Poor compliance by patients is a major barrier to getting GC/T prior to surgery. Backes et al. previously reported poor compliance with genetic counseling referral among patients with IHC results suggestive of LS [16]. This group surveyed patients about barriers to pursuing genetic counseling services and their risk perception. They found that most patients underestimated their risk of LS and associated cancers, and only 57% of patients expected to benefit from genetic counseling. Only 28% of patients in their study actually pursued genetic counseling. They reported the most frequent barrier was insurance coverage/cost. Additionally, Tomiak et al*.* found that the term “genetic counseling” was a deterrent for patients to schedule an appointment because they thought it meant they would be visiting with a psychological counselor. [17] To mitigate the perception that genetic counseling implies psychological counseling, institutions may use outreach and educational material and use terms like genetic evaluation. Increasing knowledge of LS among referring physicians may help promote referrals and alleviate any patient confusion or fears in the context of a dMMR biopsy screening result. Lastly, surgeon engagement is critical to the success of this process. Surgeons must be aware that this information is available and have open communication with the pathologist and genetic counselors. Anecdotally, patients are more likely to undergo genetic counseling and testing when their surgeon explains the importance of the test results and how it would impact treatment and diagnoses for the rest of the patient's family.

Long wait times for appointments with genetics is likely another barrier to timely assessment, as there were sometimes months between patients’ referral dates and appointment dates. Centers should consider prioritizing patients with dMMR results to an expedited appointment. Of note, one patient in our study that had their results and had GC/T prior to surgery received neoadjuvant chemotherapy. This unique circumstance allowed more time before surgery for their results to become available and to complete their visit with genetics before surgery. It would be worth keeping record of which patients will receive neoadjuvant chemotherapy when triaging results/appointments for patients. Overall, a collaborative approach is best with interdisciplinary communication, clear roles for each subspecialist, and complete explanations for patients. As suggested by Chubak et al*.*, an informational fact sheet could be provided in the patient's preoperative materials to increase awareness about MSI/IHC testing and LS [18], and we provide such a fact sheet at our institution.

Another barrier to informed preoperative counseling can occur when patients have complete germline testing prior to surgery but have still have an unexplained dMMR tumor. This can occur when MMR IHC screening suggests LS due to absent MMR protein, and genetic sequencing for MMR genes does not identify a germline mutation [19]. For these patients, tumor analysis is then necessary to detect somatic MMR mutations that would explain their dMMR tumor. Unfortunately, not all commercial laboratories accept biopsy specimens to perform this testing due to insufficient tumor quantity. Therefore, it may not be possible for patients with these discordant results to have full genetic testing results, both germline and somatic, prior to their surgery.

In one case in our study there was no mention of IHC screening results or recommendations for genetic counseling in preoperative notes by surgeons. As our study used a retrospective chart review, we were unable to know whether or not these complex preoperative discussions occurred. It is possible that surgeon bias or lack of knowledge about Lynch Syndrome and the risk of metachronous colorectal cancer may have affected decision-making, but ultimately, the patients did make the final decision. Of note, some patients suspected of having LS elected to pursue subtotal or extended colectomy while others chose a less extensive surgery after a risk/benefit discussion with their surgeon. Given the many surgical and surveillance options available to patients, an informed discussion is vital to deciding the best care for each individual patient.

We found several areas of potential process improvement to allow for more patients to have informed discussions with their surgeons prior to resection. The main area of improvement we identified is to obtain biopsies from outside institutions and screen them prior to planning the resection, as almost half of the total cases in our study had no biopsy available before resections to screen. Other areas of improvement include making sure patients get timely referrals for genetic counseling and testing, ensuring patients are not lost to follow up, shortening wait times for appointments with genetics, and improving patient compliance with making and attending appointments by providing thorough instructions and explanations of the benefits of genetic counseling prior to surgery.

## Conclusion

We analyzed the performance of UTS on biopsies rather than resection specimens and the clinical impact. Overall, we found that 21.4% of patients with dMMR CRC who underwent preoperative biopsy testing made a fully informed surgical/treatment decision based on their dMMR status/diagnosis of LS prior to resection. Although our results are from a single institution, they are the first to show the implications of UTS for LS on biopsy specimens in terms of influencing surgical decision-making. Many challenges remain in using biopsies for LS screening to help inform the surgical procedure, especially at a tertiary institution, and there is room for improvement including patient follow-up and coordinating pre-surgery genetics consultations.

## Data Availability

The datasets generated during and/or analyzed during the current study are available from the corresponding author on reasonable request.
